# Improving nutrition budgeting in health sector plans: Evidence from India's anaemia control strategy

**DOI:** 10.1111/mcn.13253

**Published:** 2022-03-01

**Authors:** Avi Saini, Ritwik Shukla, William Joe, Avani Kapur

**Affiliations:** ^1^ Institute of Economic Growth University Enclave, Delhi University, North Campus New Delhi India; ^2^ Accountability Initiative Centre for Policy Research Dharam Marg, Chanakyapuri New Delhi India; ^3^ Population Research Center (PRC), Institute of Economic Growth University Enclave, Delhi University North Campus New Delhi India

## Abstract

In India, 15 nutrition interventions are delivered and financed through the National Health Mission (NHM). Programmatic know‐how, however, on tracking nutrition budgets in health sector plans is limited. Following the four phases of the budget cycle—planning, allocations, disbursements and expenditure, this paper presents a new method developed by the authors to track nutrition budgets within health sector plans. Using the example of the Anemia Mukt Bharat (AMB) or Anemia Free India strategy, it reports preliminary findings on the application of the first two phases of the method, that is, to track and act for improved planning and allocations, covering 12 states. The paper lists out the budget heads, cost norms and developed tools to plan adequately. Supportive action was undertaken through sharing trends and trainings for AMB's budgeting to create opportunities for improvements. It was observed that the AMB budget increased over 3 years despite the COVID situation. It increased from INR 6184 million in FY 2019–2020 to INR 6293 million, a 2% increase in FY 2020–2021, and to INR 7433 million, an 18% increase in FY 2021–2022. The difference in allocations and planned budgets were low (16%, 4% and 11%, respectively) while the difference in required budgets and planned budgets were significant but reduced consistently (41%, 31% and 22%, respectively). The paper concludes that the methods adopted for tracking and acting for improved nutrition budgets helped in informing national and state governments regarding yearly trends. Such methods can be effective and be developed for other nutrition interventions.

## Key messages

- Tracking budgets and timely action can improve planning and allocation efficiency.
- Support to states in planning budgets is essential. No major gaps were found in approvals.
- Technical partners can assist in supporting governments for improving financial efficiency.

## 1 INTRODUCTION

Global and national research on nutrition interventions have found that adequate finance is one of six key health systems building blocks (World Health Organization, 2007) and critical for ensuring universal coverage of these interventions (Kapur et al., 2020; Menon et al., 2016; Shekar et al., 2017) and for programme equity (Rajan et al., 2016). Regularly tracking nutrition programme finances is necessary for governments and partners for decision‐making and to prioritize nutrition and optimally utilize allocated funds (SPRING, 2018).

Nutrition budgets are spread across multiple ministries and departments and often are integrated within other sectors or programmes (Kapur & Shukla, 2021; Lamstein et al., 2016). Hence, tracking and disaggregating budgets for nutrition are challenging. In India, 15 nutrition interventions for women and children are delivered and financed through a health programme known as the National Health Mission (NHM), implemented by the Ministry of Health and Family Welfare (MoHFW), Government of India (Ministry of Health and Family Welfare, 2013). One of these interventions is an anaemia reduction strategy called Anemia Mukt Bharat (AMB)/Anemia Free India. Using the example of the AMB strategy, this paper presents a new method developed by the authors to track nutrition budgets within health sector plans, known as the Track and Act Method. Further, the paper reports preliminary findings on the application of the first two phases of this method which are tracking and providing supportive action for informing policymakers and improving planning and allocation in 12 states.

The paper is structured as follows. The Section 1.1  gives a background on AMB. Section 2 presents the detailed methodology on the creation of the *Track and Act Method* including data sources. Section 3 presents the findings of the application of the method across 12 states. Finally, Section 4 provides a short discussion on the implications of the findings.

### 1.1 Background on AMB

Anaemia has persisted as a major public health and nutrition challenge in India (World Health Organization, 2011). As per the National Family Health Survey (2015–2016), 58.4% of children (6–59 months) and 53% of women (15–49 years) were anaemic (International Institute of Population Sciences, 2016). Despite increasing policy focus since the 1970s, anaemia prevalence has declined slowly, with considerable geographic heterogeneity (Nguyen et al., 2018). In 2018, with renewed emphasis, the union government launched AMB or Anemia Free India strategy. AMB intensified earlier approaches, aiming to improve governance architecture and encouraging convergence among ministries and departments. It was integrated with the broader agenda of nutritional health and development through POSHAN (Prime Minister's Overarching Scheme for Holistic Nourishment) Abhiyan. AMB targets reducing anaemia prevalence by 3% per annum by 2022 for various groups including children aged 6–59 months, adolescents (15–19 years), Women of Reproductive Age (WRA, 15–49 years), pregnant women and lactating mothers. (Ministry of Health and Family Welfare MoHFW, 2018). These are consistent with international commitments to anaemia reduction. For example, the World Health Assembly aims to reduce anaemia by 50% in women of reproductive age by 2025, or the Sustainable Development Goals aim for a reduction in anaemia by 2030.

AMB focuses on six interventions, that is, (i) prophylactic iron and folic acid (IFA) supplementation; (ii) deworming; (iii) intensified year‐round Behavior Change Communication (BCC) campaign; (iv) testing and treatment of anaemia using digital methods and point of care treatment; (v) mandatory provision of IFA‐fortified foods in government‐funded public health programmes; and (vi) intensifying awareness, screening, and treatment of nonnutritional causes of anaemia in endemic pockets, with a special focus on malaria, haemoglobinopathies and fluorosis.

For this, AMB operational guidelines (Ministry of Health and Family Welfare MoHFW, 2018) specified a resource envelope (INR 10.9 million per district). These funds, however, are spread across multiple components, mostly within the NHM budget.

Two features of AMB enabled testing out the Track and Act method. First, it had financial guidelines and information systems in place. Second, AMB involved multiple stakeholders at the national and state levels, enabling mid‐year course correction via technical assistance.

## 2 METHODS

Evidence on effective public finance management for nutrition (Picanyol et al., 2015) suggests following the funds through the policy cycle—planning or resource cost estimations, budgeting at scale, ensuring funds reach the last mile, and finally, that they are utilized effectively. Funds for health programmes, notably NHM, have been tracked by various authors (Ghai et al., 2016; Srivastava et al., 2017). There are two notable differences from previous work. First, the Tract and Act method tracks all components of the budget cycle from resource requirements to expenditures in real time providing insights for course corrections during the budget cycle. Second, it integrates supportive action measures like training and tools parallel to tracking budgets to create opportunities for improvement in the budgeting of specific strategies like AMB within the health sector.

### 2.1 Development of Track and Act Method

**Figure 1:**
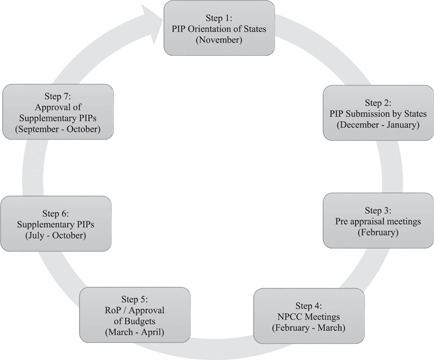
Steps in planning and approval of NHM financial plans. NPCC, National Programme Coordination Committee; PIP, Programme Implementation Plan; ROP, Record of Proceeding. *Source*: Authors based on Finance Management Group Guidelines, 2012

The method was based on NHM's planning and budgeting system. As per the design, NHM aims to follow a bottom‐up approach to planning. The block office collates village‐level plans and sends them to the district. The district then creates District Health Action Plans which are then aggregated into State Plans also called Programme Implementation Plans (PIPs), incorporating inputs from each level (Ministry of Health and Family Welfare MOHFW, 2012). These PIPs are then submitted to the MoHFW for appraisal and approval. These are appraised during the National Programme Coordination Committee (NPCC), following a discussion between officials from the state and union governments. Once approved, these are known as Record of Proceedings (RoPs) (Figure 1).

This process usually begins around November, with final approvals coming around March (end of the Indian fiscal year). NHM also provides for mid‐year course correction through supplementary budgets that can be requested from July to October (Ministry of Health and Family Welfare MOHFW, 2012).

**Figure 2:**
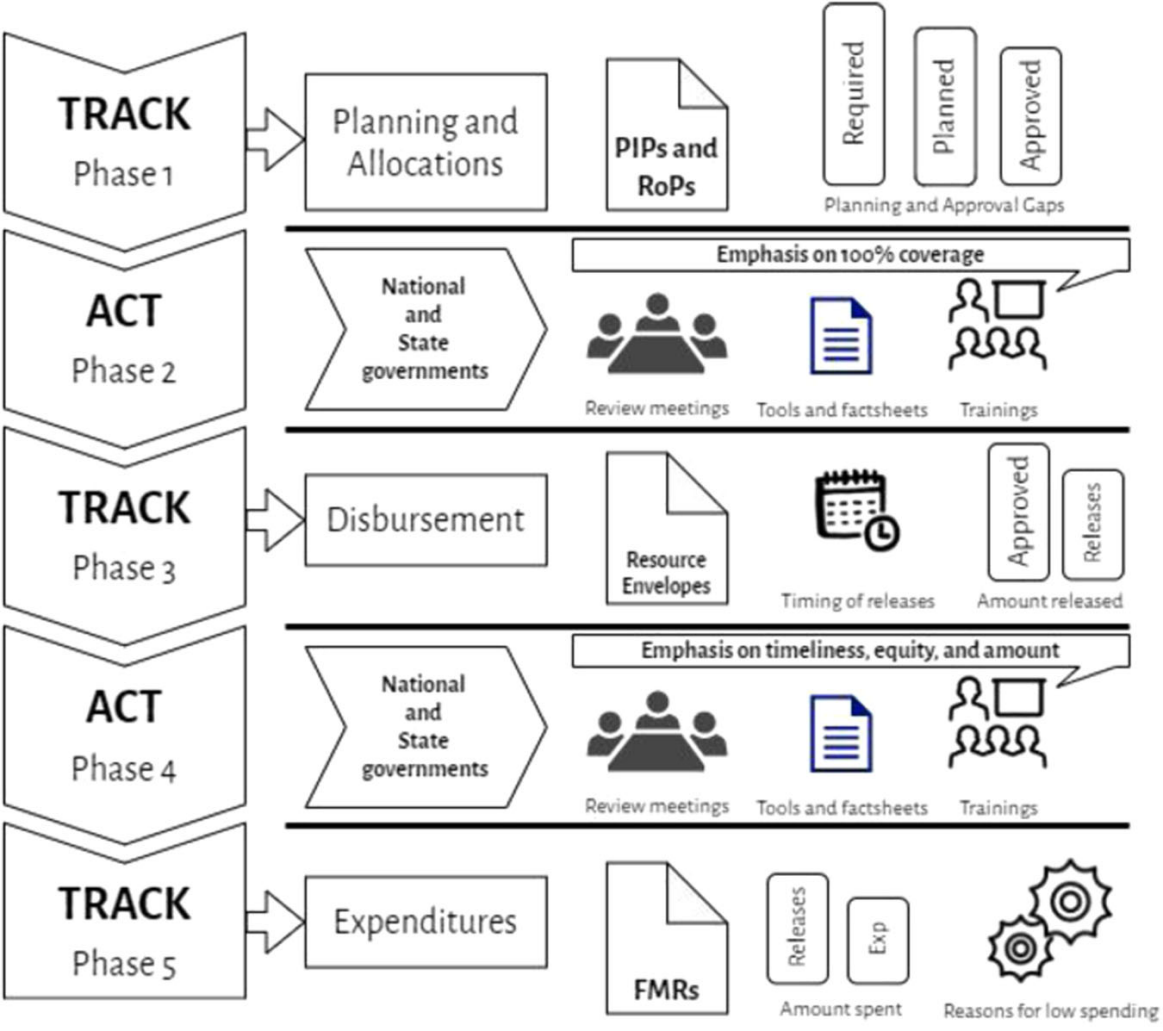
Track and Act Method. FMR, Financial Management Report; PIP, Programme Implementation Plan; ROP, Record of Proceeding. *Source*: Authors

The development and application of the Track and Act Method had five phases based on the budget cycle (Figure 2).

**Figure 3:**
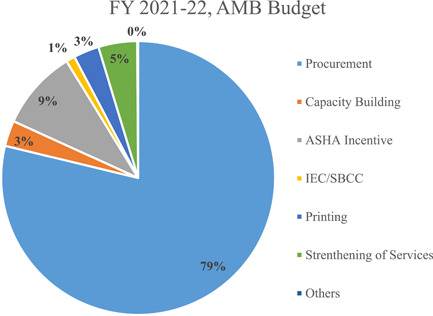
Contribution of each major budget head in the total AMB budget, 12 states, India, FY 2021–2022. Others involve program management, drug and warehouse logistics, human resources, research and innovation. *Source*: Authors based on NHM PIP and RoP documents

First, referring to the interventions mentioned in AMB guidelines, a list of AMB budget line items was collated. State‐wise planned and approved budgets were calculated against the major and minor budget heads identified. This was compared to the estimated budget required for 100% coverage to calculate differences in planning and allocations. This tracking was completed by July to enable funds requests via supplementary budgets proposed by states, that is, a mid‐course correction. The second phase involves supportive action including sharing information on planning gaps and through meetings, training and sharing budget tracking tools. This was completed by November to allow revisions in state‐level planning for the next financial year. The third phase requires assessing the disbursement of approved funds for timeliness and equity. Suggestions to improve the timeliness and equity of disbursements are shared in the fourth phase, along with developing tools for the same. Finally, the fifth stage tracks expenditures (Figure 3).

This paper reports the application of the first two phases of the method—the planning and allocation of funds for AMB. This has been done due to a lack of publicly available data on disbursements and expenditures (Kapur & Shukla 2021; Centre for Budget and Governance Accountability CBGA, 2017), and the depth of issues with planning and allocations which require focus.

### 2.2 Applying the Method on AMB

Two key data sources on NHM planning and budgeting were used: NHM PIPs and RoPs. Both documents for all states and union territories (UTs) are publicly available (Ministry of Health and Family Welfare MOHFW, 2019–2022).

Additionally, the study relied on discussions with several officers/consultants for clarifications and technical guidance. These include nutrition specialists, programme officers and AMB consultants of state teams across the 12 study states and the AMB Programme Management Unit at MoHFW. Developing and implementing the method involved five steps, outlined below:

#### 2.2.1 Tracking the budget

First, a list of budget heads to track was created. NHM PIPs (proposed budget by state) and RoPs (approved budget by MoHFW) are disaggregated into several budget heads, with specific codes known as the Financial Management Report (FMR) codes. Subactivities have minor FMR codes which are clubbed under one major FMR code (Ministry of Health and Family Welfare MOHFW, 2012). Given the lack of a consolidated AMB budget, a first step was to identify the strategy‐specific FMR codes. Based on this assessment, a total of 11 major and 46 minor eligible budget heads under the purview of AMB were identified in PIPs.

Budget heads were selected based on their relevance to each of the six interventions mentioned earlier that AMB focuses on. All budget heads related to IFA supplementation were included. For deworming and nonnutritional causes of anaemia, separate national‐level programmes are operational. Hence, only selected budget heads related to these strategies were included. For deworming, intervention budgets related to procurement, capacity building, and Information, Education, Communication/Behaviour Change Communication (IEC/BCC) were included. Similarly, for haemoglobinopathies, budgets for procurement and capacity building were included. For malaria, only the cost of insecticide‐treated bed nets was included, as it is a focus activity under AMB. For fluorosis, no budget heads which directly affect the AMB programme were found, and hence it was excluded. Further, any anaemia‐related research, innovation, human resource, drug warehouse and logistics, and programme management that was proposed or approved were included. The list of identified major and minor budget heads is presented as Appendix Table S1.

Second, the required budget was calculated by using denominators (e.g., population figures, the number of community health workers), unit costs and calculation norms for each budget head. Information on category‐wise population was sourced from the publicly available data in the AMB dashboard (an online portal for AMB, MoHFW). States report the number of target beneficiaries, and these were multiplied with unit costs to arrive at the state level budget requirements under AMB. Further, information on unit costs of drugs (IFA, albendazole, iron, sucrose, etc.), AMB training, the number of ASHAs, the number of health subcenters, the number of school health teams, details of IEC/BCC campaigns, research and innovation projects, and so forth were obtained from state‐specific PIPs and ROPs. A tool (available in Supporting Information) was developed to automatically calculate the required budget for each sample state with prefilled cost norms.

Third, the required budget was benchmarked against the planned budget and approved budget to observe differences in these. Unbudgeted components were also identified.

This process was conducted for 12 states accounting for 76% of India's AMB target population in the first phase (July to August, FY 2019). These were Assam, Bihar, Chhattisgarh, Gujarat, Jharkhand, Madhya Pradesh, Maharashtra, Odisha, Rajasthan, Telangana, Uttar Pradesh and West Bengal. These states were selected as UNICEF state teams were involved in the state planning process, providing feedback and conducting state‐level in‐depth technical analytical guidance which was integral to the method.

#### 2.2.2 Action

Fourth, the Track and Act method focused on supportive action based on insights from tracking budgets using a nonfault‐finding approach. The objective was to list out areas that needed attention and enable planners to cover components comprehensively. Therefore, an important step was to share findings with state‐level NHM officials and enable data‐based budgetary decisions. Supportive action included union and state level dissemination of the findings, the inclusion of training on financial planning using the tracker in AMB cascade trainings and national reviews, and the preparation of costing templates to support state programme managers to appropriately plan for upcoming budget cycles. Building trust and coordinating with governments was key. Other development partners were also involved in training and necessary materials were shared with them. In particular, state‐wise factsheets that highlighted gaps, items missed and items under‐budgeted were discussed/considered by MoHFW and states to ensure that these were addressed during PIP submissions and NPCC meetings for next year. Lastly, changes in planning and allocation over the period of three financial years, namely FY 2019–2020, FY 2020–2021 and FY 2021–2022 were studied.

## 3 RESULTS

This section describes the preliminary results of using the Track and Act method in observing changes in required, planned and allocated budgets across three years namely FY 2019–2020, FY 2020–2021 and FY 2021–2022, following training and supportive actions done with government stakeholders. Although the change is assessed to observe the difference in budgets across years, we are not implying causality or conducting a statistical evaluation of impact. Budgets may have changed as budgeting involves many political and administrative factors and not just the application of the track and act method.

In FY 2019–2020, the states required INR 12,432 million for universal coverage of AMB. States proposed INR 7377 million or 41% of the required budget. From this, INR 6184 million or 84% of the proposed amount was approved. Thus, overall, 50% of the required budget was available in FY 2019–2020 for programme implementation (Table 1).

**Table 1:** Estimated required, planned and allocated budget for Anemia Mukt Bharat, 12 states, India, FY 2019–2020, FY 2020–2021 and FY 2021–2022, INR in million

	Required	Planned	Planned vs. Required (%)	Allocated	Allocated vs. Planned (%)	Allocated vs. Required (%)
FY 2019–2020	12,432	7377	−41	6184	−16	−50
FY 2020–2021	9485	6546	−31	6293	−4	−34
FY 2021–2022	10,678	8350	−22	7433	−11	−30

*Note*: One million INR is equal to USD 13,634 as on September 2020.

Interestingly, as compared to FY 2019–2020, planned budgets for FY 2020–2021 all states were closer to required budgets resulting in a reduction in the gap between estimated requirements and planned budgets. For instance, the difference in planned and required budget was reduced to 31%, a decrease of 10 percentage points, which further reduced to 22% in FY 2021–2022. In FY 2019–2020, (Table 2) the maximum difference in estimated requirement and planned budget was in West Bengal (69%), Gujarat (59%) and Telangana (49%). In FY 2020–21, this difference was maximum in West Bengal (58%), Bihar (55%) and Uttar Pradesh (42%). In FY 2021–2022, this difference was maximum in the same states but the percentage points reduced namely West Bengal (47%), Bihar (37%) and Uttar Pradesh (39%).

**Table 2:** Change in estimated required budget and percentage change in planned against required for AMB, 12 states, India, FY 2019–2020, FY 2020–2021 and FY 2021–2022, INR in million

		Budget required (INR in million)			% difference between planned and required budgets		
SN	Budget heads	FY 2019–2020	FY 2020–2021	FY 2021–2022	FY 2019–2020	FY 2020–2021	FY 2021–2022
1	Assam	364	374	438	−37	−20	−16
2	Bihar	1727	1550	1274	−43	−55	−37
3	Chhattisgarh	573	514	391	−20	−3	−15
4	Gujarat	782	318	378	−59	−15	−1
5	Jharkhand	540	496	635	−16	−11	−10
6	Madhya Pradesh	989	834	1281	−8	4	29
7	Maharashtra	1398	674	1147	−32	−9	−3
8	Odisha	364	226	338	−39	−27	−10
9	Rajasthan	978	721	575	−43	−31	−35
10	Telangana	641	717	526	−49	−5	−36
11	Uttar Pradesh	2455	1464	1891	−41	−42	−39
12	West Bengal	1622	1596	1805	−69	−58	−47
	Total	12,432	9485	10,678	−41	−31	−22

*Note*: One million INR is equal to USD 13,634 as on September 2020.

In FY 2019–2020, a total of INR 6184 million was allocated for AMB in 12 study states. In FY 2020–2021, INR 6293 million was allocated, a 2% increase. Out of 12 states, the budget increased in seven states with the highest increase in Telangana (419%), followed by Assam (48%) and Chhattisgarh (33%). Five states were allocated lower budgets than the previous year with the highest decreases observed in Uttar Pradesh (36%), followed by Maharashtra (28%) and Odisha (22%).

In FY 2021–2022, INR 7433 million was allocated, 18% more than the previous year. Out of 12 states, the budget increased in 8 states with the highest in Madhya Pradesh (82%), followed by Odisha (75%) and Maharashtra (51%). Four states were allocated a lower budget against the previous year. This included in descending order Telangana (46%), followed by Rajasthan (28%) and Chhattisgarh (24%).

Coincidentally, FY 2019–2020 preceded the COVID‐19 pandemic, and FY 2020–2021 and FY 2021–2022 were during the pandemic. This perspective has been used in the  “Discussion” section.

Out of 11 major budget heads or components of AMB, procurement remains the biggest component (79%) in the overall AMB budget, followed by incentives for community workers (9%). The contribution of critical components like capacity building, IEC/BCC, printing and strengthening of services were low, that is, 3%, 1%, 3% and 5%, respectively. Research and Innovation remain negligible in the AMB budget (Table 3).

**Table 3:** Percentage change in allocated budget of AMB across 12 states, India, FY 2019‐20, FY 2020‐21 and FY 2021‐22, INR in million

		Budget allocated				
		FY 2019–2020	FY 2020–2021	% Change	FY 2021–2022	% Change
1	Assam	201	298	↑48	347	↑16
2	Bihar	690	690	↑0	780	↑13
3	Chhattisgarh	322	427	↑33	325	↓24
4	Gujarat	317	268	↓15	376	↑40
5	Jharkhand	352	432	↑23	510	↑18
6	Madhya Pradesh	907	841	↓7	1527	↑82
7	Maharashtra	823	593	↓28	895	↑51
8	Odisha	212	166	↓22	291	↑75
9	Rajasthan	463	499	↑8	359	↓28
10	Telangana	120	623	↑419	337	↓46
11	Uttar Pradesh	1271	814	↓36	1152	↑42
12	West Bengal	507	641	↑26	534	↓17
	Total	6184	6293	↑2	7433	↑18

*Note*: One million INR is equal to USD 13634 as on September 2020.

Further analysis into components showed that over the 3 years, procurement received the highest increase namely by 6% (INR 259 million) in FY 2020–2021 and then 29% (INR 1330 million) in FY 2021–2022. The budget allocated for IEC/BCC (by INR 104 million) and printing (by INR 345 million) has decreased consistently in this period (Table 4).

**Table 4:** Component wise allocated budget for Anemia Mukt Bharat, 12 states, India, FY 2020–2021 and FY 2021–2022, INR in million

	FY 2019–2020	FY 2020–2021	% Change	FY 2021–2022	% Change
Procurement	4266	4525	↑6	5855	↑29
Capacity building	215	227	↑6	225	↓1
ASHA incentive	627	669	↑7	701	↑5
IEC/SBCC	182	137	↓25	78	↓43
Printing	569	253	↓56	224	↓11
Strengthening of services	306	472	↑54	341	↓28
Drugs and warehouse	6	0	↓100	0	0
Programme management	0	0	0	0	0
HR/SCEARA	1	1	0	1	0
Research	1	2	↑100	0	↓100
Innovation	13	6	↓54	8	↑33
	6184	6293	↑2	7433	↑18

*Note*: One million INR is equal to USD 13,634 as on September 2020.

Planned and allocated budgets were compared to understand the possible reasons for any significant differences. In FY 2019–2020, states had planned for INR 7377 million and had been allocated 84% of the planned budget. The highest difference in the planned and allocated budget was observed in Telangana (63%) followed by Bihar (30%) and Chhattisgarh (30%).

In FY 2020–2021, though the planned budget decreased by 11%, (INR 6546 million) from the previous year, allocations stood at 96% of the planned budget. The highest difference between planned and allocated budgets was observed in Chhattisgarh (14%) followed by Telangana (8%) and Uttar Pradesh (4%).

In FY 2021–2022, the planned budget was 28% (INR 8350 million) more than the previous year while 89% was allocated. The highest difference in the planned and allocated budget was observed in West Bengal (45%), followed by Maharashtra (19%) and Jharkhand (11%).

In terms of inclusion of all components in the AMB budget, in FY 2020–2021, 10 out of 12 states included at least one new component in their AMB budgets. For example, Jharkhand, Odisha, Maharashtra and Madhya Pradesh proposed funds for IFA red tablets for women in the reproductive age group. Bihar, Gujarat, Maharashtra, Telangana and West Bengal proposed funds for haemoglobino meters and/or consumables in FY 2020–2021. Jharkhand, Madhya Pradesh, Odisha, Telangana and West Bengal proposed funds for IEC/BCC activities in FY 2020–2021. Other components that were added by different states are ASHA incentives for mobilizing lactating women for Test, Talk and Treat (T3) camps within IEC/BCC, screening of pregnant women and school children for blood disorders, and innovation in service delivery (Tables 5, 6, 7).

**Table 5:** Change in planned budget and percentage change in allocated against the planned budget of AMB across 12 states, India, FY 2019–2020, FY 2020–2021 and FY 2021–2022, INR in million

		Planned budget			% difference between allocated and planned budget		
	States	FY 2019–2020	FY 2020–2021	FY 2021–2022	FY 2019–2020	FY 2020–2021	FY 2021–2022
1	Assam	229	298	367	−12	0.0	−6
2	Bihar	984	690	808	−30	0.0	−3
3	Chhattisgarh	460	499	332	−30	−14	−2
4	Gujarat	323	271	376	−2	−0.9	0.0
5	Jharkhand	453	442	572	−22	−2	−11
6	Madhya Pradesh	913	863	1655	−1	−3	−8
7	Maharashtra	952	612	1109	−14	−3	−19
8	Odisha	221	166	303	−4	0	−4
9	Rajasthan	560	501	371	−17	−0.4	−3
10	Telangana	326	680	337	−63	−8	0
11	Uttar Pradesh	1448	850	1155	−12	−4	0
12	West Bengal	508	674	964	0	−5	−45
	Total	7377	6546	8350	**−16**	**−4**	**−11**

**Table 6:** Number of minor budget heads covered by 12 states, India

Major heads	A	B	C	D	E	F	G	H	I	J	K	All (2021−2022)	All (2020−2021)	All (2019−2020)
Count of minor items	16	7	3	4	4	6	2	1	1	1	1	46	46	46
Assam	13	2	2	1	2	2	0	0	0	2	0	24	27	19
Bihar	6	2	2	1	3	2	0	0	0	0	0	16	15	24
Chhattisgarh	9	4	3	2	1	4	0	0	1	0	0	24	31	13
Gujarat	12	2	2	0	1	3	0	0	0	0	0	20	20	26
Jharkhand	8	3	2	3	4	4	0	0	0	1	0	25	32	26
Madhya Pradesh	16	2	2	1	2	0	0	0	0	1	0	24	25	20
Maharashtra	7	3	2	2	3	3	0	0	0	0	0	20	27	30
Odisha	10	3	0	0	2	2	0	0	0	0	0	17	16	17
Rajasthan	8	2	2	1	2	1	0	0	0	0	0	16	26	26
Telangana	12	3	1	1	1	2	0	0	0	0	0	20	20	14
Uttar Pradesh	10	2	1	0	2	1	0	0	0	0	0	16	24	23
West Bengal	9	4	1	0	2	1	0	0	0	0	0	17	13	10

*Note*: A—procurement; B—capacity building; C—ASHA incentive; D—IEC/BCC budget; E—M&E and Printing; F—strengthening of service delivery; G—program management; H—drug and warehouse; I—SCEARA/HR; J—Innovations; K—Research/Survey.

**Table 7:** Planned and allocated budget for Anemia Mukt Bharat, 36 states/UTs, India, FY 2019–2020, FY 2020‐2021 and FY 2021–2022, INR in million

	Planned	Approved	% Planned vs. Approved
FY 2019–2020	9784	8348	−15
FY 2020–2021	9291	8910	−4
Change %	↓5	↑7	
FY 2021–2022	12,837	11,642	−9
Change %	↑38	↑31	

In FY 2021–2022, the components proposed were reduced in a few states because the fixed/nonrecurring activities were completed such as procurement of haemoglobino meters, training etc. and printing reduced. Also, due to the COVID‐19 pandemic, certain community‐based IEC/BCC, training and research activities were not planned.

The above observations were shared with MOHFW regularly in FY 2019–2020, and as a result, the team was requested by the concerned division to share the planned and approved budget for all 36 states and UTs from then onwards. Similar to the trends for these 12 states, there was a consistent improvement in the overall AMB allocations for all 36 states and UTs. In FY 2020–2021, INR 8910 million was allocated which was a 7% increase against the previous year's allocation. Further, in FY 2021–2022, INR 11,642 million was allocated, a 31% increase.

Though the planned budget was 5% lower budget in FY 2020–2021 compared to the previous year, 96% of what was planned was approved by MoHFW. In FY 2021–2022, however, the planned budget was 38% higher than the previous year and about 91% of this budget was approved. State‐wise details are available in Appendix S2.

## 4 DISCUSSION AND CONCLUSION

The four salient results from the application of the first two phases of the Track and Act method for the 12 selected states are as follows. First, the difference in the required budget for 100% coverage of beneficiaries and planned budget by states was estimated to be 41% in FY 2019–2020. It reduced to 31% in FY 2020–2021 and to 22% in FY 2021–2022. Second, there were improved funds available for AMB within NHM approved plans throughout the 3 year period. The budget was increased in FY 2020–2021 and FY 2021–2022 by 2% and 18%, respectively. This period witnessed the COVID‐19 pandemic period. Despite that, budgets for AMB increased even as health funds were routed to meet the COVID‐19 response effort. Third, most of the planned budget was approved in all 3 years (FY 2019–2020, 86%; FY 2020–2021, 96%; and FY 2021–2022, 89%). Fourth, it was noted that timely and systematic exposure to research findings with key stakeholders can potentially act as an important catalyst to support union and state governments in creating opportunities in improving nutrition budgeting within health sector financial plans.

Before discussing these findings, it is worth noting the key limitations of this analysis. First, certain items were excluded from the analysis of required and planned budgets. For example, goods and services volunteered or provided by nongovernmental agencies (NGOs) and partners were excluded. These include items such as IFA tablets and syrup, or services such as filling gaps in human resources and capacities by supporting government activities. Second, states may have contributed actively to reduce anaemia via other state‐funded activities and programmes. Therefore, this analysis accounts for items such as these for which information is available. For example, in FY 2020–2021, Odisha procured IFA supplements through the state drug corporations whereas Assam procured haemoglobino meters through the state budget. Both of these are mentioned in RoPs and have been accounted for by subtracting the amount from the required budget. Similarly, Gujarat procured IFA via union government funds directly received as part of central procurement. This has also been accounted for while estimating requirements. Third, for some major heads, such as programme management, drug and warehousing, human resources, states spend from the budget for routine NHM activities. Since demarcating such funds is difficult, these heads were included only when any explicit reference was made in the RoPs. Fourth, norms and standards for some major heads such as IEC/BCC, printing and strengthening of service delivery were unavailable. Therefore, the amounts proposed by states have been treated as required amounts. The same is true for research and innovation. By choosing the proposed amount as a proxy for the required amount, we may potentially underestimate planning and approval gaps. However, these components account for only 7% of the required amount, and therefore any underestimations are small. Fifth, the Track and Act methodology may not necessarily have a causal association with improvements in budgeting practices and increased budgets. There are several other parameters such as interest, motivation and focus of various stakeholders as well as the broader policy attention on anaemia prevalence that may shape budgets. Lastly, while the present findings only speak to the first two phases, there may also be differences between approvals and disbursements, and disbursements and expenditures.

Despite these limitations, the results confirm that despite a comprehensive planning architecture within NHM, there were differences between budgets required (to achieve 100% coverage of programme targets) and those proposed and finally approved for AMB across most states. Based on the findings, regular feedback was provided to union and state governments through tools, review meetings and AMB cascade trainings.

Six important insights emerge from the analysis:

First, a complete list of budget heads along with cost norms is critical for ensuring adequate planning and budgeting. The AMB operational guidelines, released in 2018, mention six major budget heads namely (a) procurement, (b) incentive for community health workers (ASHAs), (c) IEC/BCC activities, (d) monitoring and evaluation, (e) capacity building and (f) miscellaneous. Even in terms of minor heads, the guidelines lay out 19 minor heads compared to the 46 identified in the study.

Second, resource caps limited the budgeting scale at both state and union levels. This meant that even if states recognized the overall requirements, they had limited space to propose them due to resource caps for the programme.

Third, while the analysis examined the difference in requirements and planned budgets, in practice it is also important to recognize that states may have different objectives while planning. States where the coverage of key programme indicators was poor usually proposed lower and probably more achievable targets. It is possible that the reasons for low coverage vary state‐wise, and can be driven by factors like or delayed procurement of supplies on account of supply chain management, lack of human resources, the non‐rollout of several components for WRA (women in reproductive age), and so forth, all of which support program delivery. Such states may plan on increasing coverage gradually over time and may budget accordingly.

Fourth, the COVID‐19 pandemic did not lead to a decline in planned and allocated budgets for AMB. The only component that might have been majorly affected was IEC/BCC. Its allocations declined by 41% in FY 2021–2022. However, the situation might have affected states differently and might not reflect in overall amounts. As per RoP comments, a few states such as Jharkhand and Chhattisgarh reported being unable to utilize funds for procurement of supplements as coverage dropped drastically. This led to unutilized stock and hence reduced planned budgets accordingly. Few states had to repropose activities that did not get completed in FY 2020–2021 such as procurement of haemoglobino meters due to lockdown, leading to an increase in allocated budgets. States need to plan strategically to improve investments in components like IEC/BCC, capacity building, research and innovation, especially in times like the COVID‐19 pandemic.

Fifth, in both FY 2020–2021 and FY 2021–2022, budget planning and approvals for AMB improved. States proposed funds for more components, as opposed to FY 2019–2020. Bihar proposed funds for seven new components, while Jharkhand, Telangana, West Bengal and Madhya Pradesh proposed funds for four more components. Odisha,  Maharashtra, Chhattisgarh, Gujarat, Assam and Rajasthan proposed funds for more than one new component each. States also accounted for more beneficiaries while planning. While all the 12 states increased the population budgeted for by a certain percentage, Madhya Pradesh proposed more than the required amount. However, it is important to note that the total required budget in FY 2020–2021 for these states was about 25% less than the required budget in FY 2019–2020. This is attributable to the reduction of fixed costs and unspent balances from the previous year. For instance, by FY 2020–2021, one‐time activities like the orientation of state and district officials had been completed in most states. Similarly, the procurement of digital haemoglobino meters was partially completed in many states. At the same time, the requirement for IFA and Albendazole was reduced due to available stock or unspent balance from FY 2019–2020.

Later in FY 2021–2022, the requirement increased due to two key reasons. First, six states namely Assam, Bihar, Madhya Pradesh, Odisha, Uttar Pradesh and Maharashtra improved coverage of services like IFA supplementation, haemoglobino meters, ASHA incentive, capacity building, and drugs and supplies for haemoglobinopathies. Second, the unspent balance and stock available from FY 2019–2020 reduced and states proposed budgets accordingly.

These findings suggest that while there are several factors constraining planning and budgeting, regularly tracking and exposure/engagement to improve plans and budgets can provide a useful benchmark to states and decision‐makers to understand exact requirements.

Sixth, regularly sharing research findings with key stakeholders in a timely manner can act as an important catalyst for improving financial efficiency. In FY 2020–2021, cognizant of the improvement made in states, the union government directed the national team to replicate the planning and allocations part of the method for all 24 remaining states/UTs, with a focus on approval gaps. Additionally, a chapter on PIP support was included in the national AMB training toolkit which details suggestions for accurate cost estimations and budgeting efficiently. Moreover, the method was applied for maternal nutrition components which were well received by MoHFW. Although it is not conclusive that the changes in the AMB budget were due to only the tracking and supportive actions, such efforts of creating regular feedback mechanisms can be of assistance to strengthen fiscal efficiency.

It is important to understand that improved planning and approvals, though critical, may not immediately lead to improvements in programme coverage. Currently, the comparison of coverage of AMB's key performance indicators (such as IFA provision) and the planning and approval budget gaps in FY 2019–2020 and FY 2020–2021 shows no direct relation.

Moreover, the financial cycle includes two more important stages, that is, disbursements and expenditures which are part of the Track and Act method. What money actually gets spent by whom, on what items, and for what purpose is often determined during the process of budget execution, which in itself implies political, financial and technical interactions within a basket of interests and powers (Rajan et al., 2016).

The designing and implementation of the Track and Act method and the consequent findings point to several recommendations that can further strengthen the programme. First, the availability of financial guidelines, unit costs and denominators for AMB supported analyses of fiscal gaps. Data can be used effectively to assist a key block of implementation—adequate financing. To this end, disaggregated financial data (proposed, approved and utilized funds) should be made publicly available every year. Second, the creation of clear and precise toolkits can enable uptake as well. The tool can inform program managers at the union/state level on planning and allocative efficiency and help address gaps in subsequent years. As NHM encourages bottom to top approach for planning, technical assistance should be provided up to the district level to ensure need‐based planning. Tools and training developed for AMB as part of the Track and Act method can be useful templates for engaging district‐level planners. Third, fiscal planning training for program planners and development partners supported validation of the existence of gaps and built a critical mass of support to improve planning referring to this study. Avoiding a fault‐finding approach and adopting an appreciative approach with the government is useful. Lastly, the track and act approach can be widely applied across different programmes, developing countries that follow line‐item budgeting (Piatti‐Fünfkirchen & Schneider 2018), and types of budget cycles. The fundamentals are tracking, and engaging decision‐makers with ‘acting’, which are generalizable.

The future of AMB and many new and old initiatives in India's nutrition landscape hinges on several crucial factors, of which financing is one. Therefore, while concluding, it is worth reiterating the crucial role of developmental and technical support partners in the current policy landscape to assist states in improving nutrition financing. This analysis presents encouraging evidence on supporting governments, including sharing findings of such studies in a cogent and constructive manner. The application of this method to other interventions and other components of financial efficiency (disbursements and expenditures) and assessing gaps filled by state schemes is required and is underway. These findings, however, are relevant for assessing the utility of the Track and Act method.

## CONFLICT OF INTERESTS

The authors declare that there are no conflict of interests.

## ETHICS STATEMENT

No ethical approval required as the data were sourced from an open access website.

## AUTHOR CONTRIBUTIONS

AS and WJ visualized, conceptualized and designed the study. AS performed the research and analysed the data. RS and AS wrote the paper. AK and WJ reviewed and edited the paper.

## Supporting information

Supporting information.

## Data Availability

The datasets were derived from sources in the public domain. National Health Mission website, Ministry of Health and Family Welfare, Government of India (https://nhm.gov.in/index4.php?lang=1%26level=0%26linkid=449%26lid=53).
