# Supplementary material for: Improving nutrition budgeting in health sector plans: Evidence from India's anaemia control strategy
Source: Matern Child Nutr. 2022 Mar 1;18(2):e13253. doi: 10.1111/mcn.13253 (PMC8932726; doi:10.1111/mcn.13253)
Supplement: Supplementary file 1 — Supporting information. [file MCN-18-e13253-s001.docx]

**Table S1: List of selected AMB budget heads**

| **FMR Code** | **Budget Heads** |
| --- | --- |
|  | **PROCUREMENT** |
| 6.2.1.5 | IFA Tablet for non-pregnant & non-lactating women in reproductive Age (20-49 Yrs) |
| 6.2.1.7.a | IFA Tablet for Pregnant & Lactating Mothers |
| 6.2.1.7.b | Folic Acid Tablets (400mg) for pregnant mothers |
| 6.2.2.3 | IFA Syrup (with Auto-dispenser) for Children 6-60 Months |
| 6.2.2.5 | IFA Tablet (IFA-WIFS Junior Tablets - Pink Sugar Coated) for Children 5-10 Years |
| 6.2.4.1 | IFA Tablets under WIFS (10-19 Years) |
| 6.2.1.8. | Procurement of Iron Sucrose and FCM |
| 6.2.1.6 | Albendazole Tablet for non-pregnant & non-lactating women in reproductive Age (20-49 Yrs) |
| 6.2.1.7.d | Albendazole Tablets (for pregnant women) |
| 6.2.2.4 | Albendazole Tablets for children 12-60 Months |
| 6.2.2.6 | Albendazole Tablets for children for 5-10 Years |
| 6.2.4.2 | Albendazole Tablets for children for 10-19 Years |
| 6.2.2.2. | Drugs and Supplies for NIPI and National Deworming Day |
| 6.1.1.2.2 | Any other equipment( One digital hemoglobinometer per RBSK Team and One at each Sub-centre)/ testing strip) |
| 6.2.2.9 | Any other Drugs & Supplies (Consumable for haemoglobinometer) |
| 6.2.7.2 | Drugs and Supplies for Blood related Disorders- Haemoglobinopathies |
|  | **CAPACITY BUILDING** |
| 9.5.2.3 | Orientation activities on vitamin A supplementation and Anemia Mukt Bharat Programme |
| 9.5.2.23 | One day Orientation of frontline workers (ASHA/ANM) and allied department workers (Teachers/AWW) on Anemia Mukt Bharat strategy. As per RCH training norms |
| 9.5.2.24 | Other Child Health trainings (please specify) District Level Orientation on AMB officials from Health, education ,social welfare and other allied departments |
| 9.5.2.19 | Orientation on National Deworming Day |
| 9.5.4.9 | WIFS trainings (District) |
| 9.5.4.10 | WIFS trainings (Block) |
| 9.5.6.2 | Training for Haemoglobinopathies |
|  | **Community Interventions** |
| **3.1** | **ASHA INCENTIVES** |
| 3.1.1.1.8 | NIPI incentive for mobilizing WRA (non-pregnant & non-lactating women 20-49) |
| 3.1.1.1.9 | NIPI incentive for mobilizing children and/or ensuring compliance and reporting (6-59 months) |
| 3.1.1.1.10 | National Iron Plus Others (*Lactating mothers) |
|  | **IEC/SBCC** |
| 11.4 | IEC/BCC activities under MH |
| 11.5 | IEC/BCC activities under CH |
| 11.6 | IEC/BCC activities under FP |
| 11.7 | IEC/BCC activities under AH |
|  | **Monitoring, evaluation and miscellaneous activities/Printing activities under CH** |
| 12.2.3 | Printing for Micronutrient Supplementation Programme including IEC materials, reporting formats, guidelines / training materials etc. **(For AMB and Vitamin A supplementation programmes)** |
| 12.2.5 | Printing of Compliance cards and reporting formats for National Iron Plus initiative for 6-59 months age group and for 5-10 years age group |
| 12.2.6 | Printing of IEC materials and reporting formats etc for National Deworming Day |
| 12.4.2 | Printing under WIFS -WIFS cards, WIFS registers, reporting format etc |
|  | **Strengthening Service Delivery** |
|  | **Facility Based** |
| 1.1.1.4 | Antenatal screening of all pregnant women coming to the facilities in their first trimester for sickle cell trait, Beta Thalassemia, Haemoglobin variants esp. Haemoglobin E and Anemia |
|  | **Community Based** |
| 2.3.1.2 | Line Listing and Follow up of severely anemic women |
| 2.3.1.3 | Line Listing women with blood disorder |
| 2.3.1.4 | Follow up mechanism for severely anemic women and women with Blood disorders |
|  | **School Level** |
| 2.3.3.1 | One time screening to identify the carriers of sickle cell trait , Beta Thalassemia, Haemoglobin variants at school especially class 8 students |
|  | Preventive Strategies for Malaria |
| **3.2.5.1.3/3.1.1.4.1** | **Operational cost for impregnation of Bed nets** |
|  | **Research and Surveys** |
| 10.2.1 | Research, Studies and Analysis (State specific) |
|  | **Drug Warehouse and Logistics** |
| 14.2.13 | Any other (Specify) Transportation Cost of IFA Tablets |
|  | **Programme Management** |
| 16.1.2.1.3 | Review/Orientation meeting for child Health Programmes |
| 16.1.3.3.3 | Mobility Support for DPMU/District |
|  | **State Level HR under RMNCH+A and HSS** |
| 16.4.1.3.2 | Consultant/Programme officers/HR for State Center of Excellence |
|  | **Innovations** |
| 1 | (State specific) |

**Table S2:** Percentage change in planned and allocated budget of AMB across 36 States/UTs, India FY 2019-20, FY 2020-21 and FY 2021-22, INR in millions

| SN |  | Budget Planned | | | | | Budget Approved | | | | | % Allocation vs planned | | | |
| --- | --- | --- | --- | --- | --- | --- | --- | --- | --- | --- | --- | --- | --- | --- | --- |
|  | States | FY 2019-20 | FY 2020-21 | % Change | FY 2021-22 | % Change | FY 2019-20 | FY 2020-21 | % Change | FY 2021-22 | % Change | | FY 2019-20 | FY 2020-21 | FY 2021-22 |
| 1 | A & N Islands | 5 | 3 | ↓35 | 5 | ↑47 | 4 | 3 | ↓28 | 5 | ↑43 | | -10 | 0 | -3 |
| 2 | Andhra Pradesh | 176 | 248 | ↑41 | 1427 | ↑476 | 183 | 270 | ↑48 | 1420 | ↑426 | | 4 | 9 | 0 |
| 3 | Arunachal Pradesh | 70 | 53 | ↓25 | 43 | ↓19 | 70 | 53 | ↓25 | 43 | ↓19 | | 0 | 0 | 0 |
| 4 | Assam | 229 | 298 | ↑30 | 367 | ↑23 | 201 | 298 | ↑48 | 347 | ↑16 | | -12 | 0 | -6 |
| 5 | Bihar | 984 | 690 | ↓30 | 808 | ↑17 | 690 | 690 | ↑0 | 780 | ↑13 | | -30 | 0 | -3 |
| 6 | Chandigarh | 0 | 0 | ↓100 | 1 | 0 | 0 | 0 | ↓100 | 0 | 0 | | 0 | 0 | -96 |
| 7 | Chhattisgarh | 460 | 499 | ↑8 | 332 | ↓33 | 322 | 427 | ↑33 | 325 | ↓24 | | -30 | -14 | -2 |
| 8 | Daman & Diu + Dadra & Nagar Haveli | 6 | 4 | ↓45 | 4 | ↑14 | 5 | 4 | ↓22 | 4 | ↑14 | | -29 | 0 | 0 |
| 9 | Delhi | 205 | 170 | ↓17 | 146 | ↓14 | 145 | 121 | ↓17 | 130 | ↑8 | | -29 | -29 | -11 |
| 10 | Goa | 13 | 19 | ↑43 | 13 | ↓31 | 12 | 9 | ↓28 | 3 | ↓63 | | -5 | -52 | -75 |
| 11 | Gujarat | 323 | 271 | ↓16 | 376 | ↑39 | 317 | 268 | ↓15 | 376 | ↑40 | | -2 | -1 | 0 |
| 12 | Haryana | 92 | 236 | ↑157 | 228 | ↓4 | 80 | 227 | ↑182 | 217 | ↓4 | | -13 | -4 | -5 |
| 13 | Himachal Pradesh | 69 | 79 | ↑14 | 24 | ↓69 | 62 | 79 | ↑27 | 24 | ↓69 | | -10 | 0 | 0 |
| 14 | Jammu & Kashmir | 183 | 192 | ↑5 | 226 | ↑18 | 109 | 192 | ↑77 | 218 | ↑13 | | -41 | 0 | -4 |
| 15 | Jharkhand | 453 | 442 | ↓2 | 572 | ↑29 | 352 | 432 | ↑23 | 510 | ↑18 | | -22 | -2 | -11 |
| 16 | Karnataka | 327 | 325 | ↓0 | 369 | ↑13 | 291 | 325 | ↑12 | 334 | ↑3 | | -11 | 0 | -9 |
| 17 | Kerala | 178 | 165 | ↓8 | 524 | ↑218 | 166 | 165 | ↓1 | 449 | ↑173 | | -7 | 0 | -14 |
| 18 | Ladakh | 0 | 10 | 0 | 10 | ↓5 | 0 | 10 | 0 | 9 | ↓8 | | 0 | 0 | -3 |
| 19 | Lakshadweep | 1 | 1 | ↑79 | 2 | ↑41 | 1 | 1 | ↑132 | 2 | ↑41 | | -23 | 0 | 0 |
| 20 | Madhya Pradesh | 913 | 863 | ↓5 | 1655 | ↑92 | 907 | 841 | ↓7 | 1527 | ↑82 | | -1 | -3 | -8 |
| **21** | Maharashtra | 952 | 612 | ↓36 | 1109 | ↑81 | 823 | 593 | ↓28 | 895 | ↑51 | | -14 | -3 | -19 |
| 22 | Manipur | 36 | 38 | ↑5 | 25 | ↓35 | 35 | 35 | ↓2 | 13 | ↓62 | | -2 | -8 | -46 |
| 23 | Meghalaya | 83 | 52 | ↓37 | 71 | ↑36 | 67 | 48 | ↓28 | 70 | ↑45 | | -19 | -8 | -1 |
| 24 | Mizoram | 27 | 13 | ↓53 | 30 | ↑135 | 26 | 13 | ↓50 | 16 | ↑26 | | -6 | 0 | -46 |
| 25 | Nagaland | 46 | 28 | ↓39 | 21 | ↓24 | 42 | 25 | ↓40 | 18 | ↓31 | | -9 | -10 | -18 |
| 26 | Odisha | 221 | 166 | ↓25 | 303 | ↑83 | 212 | 166 | ↓22 | 291 | ↑75 | | -4 | 0 | -4 |
| 27 | Puducherry | 33 | 20 | ↓39 | 21 | ↑5 | 21 | 17 | ↓19 | 20 | ↑14 | | -35 | -14 | -6 |
| 28 | Punjab | 302 | 251 | ↓17 | 354 | ↑41 | 302 | 248 | ↓18 | 342 | ↑38 | | 0 | -2 | -3 |
| 29 | Rajasthan | 560 | 501 | ↓11 | 371 | ↓26 | 463 | 499 | ↑8 | 359 | ↓28 | | -17 | 0 | -3 |
| 30 | Sikkim | 7 | 7 | ↑5 | 4 | ↓39 | 5 | 4 | ↓29 | 3 | ↓8 | | -24 | -49 | -23 |
| 31 | Tamil Nadu | 387 | 637 | ↑65 | 757 | ↑19 | 378 | 610 | ↑62 | 694 | ↑14 | | -2 | -4 | -8 |
| 32 | Telangana | 326 | 680 | ↑109 | 337 | ↓50 | 120 | 623 | ↑419 | 337 | ↓46 | | -63 | -8 | 0 |
| 33 | Tripura | 72 | 58 | ↓20 | 30 | ↓48 | 72 | 38 | ↓46 | 27 | ↓29 | | -1 | -33 | -8 |
| 34 | Uttar Pradesh | 1448 | 850 | ↓41 | 1155 | ↑36 | 1271 | 814 | ↓36 | 1152 | ↑42 | | -12 | -4 | 0 |
| 35 | Uttarakhand | 90 | 137 | ↑52 | 152 | ↑11 | 89 | 122 | ↑37 | 148 | ↑21 | | -1 | -11 | -3 |
| 36 | West Bengal | 508 | 674 | ↑33 | 964 | ↑43 | 507 | 641 | ↑26 | 534 | ↓17 | | 0 | -5 | -45 |
|  | **Total** | **9784** | **9291** | **↓5** | **12837** | **↑38** | **8348** | **8910** | **↑7** | **11642** | **↑31** | | **-15** | **-4** | **-9** |
